# Design Practices for Data Dashboards in Health Care: Scoping Review

**DOI:** 10.2196/77361

**Published:** 2026-02-25

**Authors:** Heike Vornhagen, Stephen Barrett, Ciara Carroll, Lydia Kavochi Iladiva, Gregory Martin, Declan McKeown, Jennifer Martin

**Affiliations:** 1 Insight Research Ireland Centre for Data Analytics Data Science Institute Ollscoil na Gaillimhe – University of Galway Galway Ireland; 2 Health Service Executive Dublin Ireland; 3 Department of Epidemiology & Public Health University College Cork Cork Ireland

**Keywords:** data visualization, dashboard design, health care analytics, evidence-based design, health informatics

## Abstract

**Background:**

Health care dashboards have the potential to enhance understanding, decision-making, and communication. However, their design, implementation, and evaluation are often hindered by the absence of standardized guidelines. This scoping review synthesizes international evidence to identify common practices for health care dashboard design, providing a foundation for application in the Irish context.

**Objective:**

This study aimed to identify existing guidelines and common practices for health care dashboard design to inform future development and implementation within the Irish health care system.

**Methods:**

A scoping review using an evidence summary approach was conducted. PubMed, Embase, Scopus, and IEEE Xplore (2014-2024) were searched. Practices were extracted and analyzed using reflexive thematic analysis and then grouped into 4 main pillars: approach (engagement of end users and stakeholders), content (data quality, effective insights, and presentation), behavior (usability and accessibility), and adoption (sustainability).

**Results:**

From 1644 initially identified studies, 18 (1.1%) met the inclusion criteria. Most were hospital focused (13/18, 72.2%), with few community- or public-facing dashboards. Only 4 of 18 (22.2%) studies described structured guidelines; most implementations (14/18, 77.8%) were ad hoc. Common practices included user involvement, actionable metrics, data quality, usability, and workflow integration. Divergences were observed: hospitals prioritized clinical indicators, public dashboards emphasized transparency, and community dashboards were underrepresented. Conflicting findings included debate over interactivity vs static simplicity.

**Conclusions:**

Dashboard design remains fragmented, with limited guidance for structured design or implementation. The 4 pillars provide a practical synthesis of best practices, highlighting pathways for evidence-informed, user-centered design. These pillars will inform future consensus building and co-design of health care dashboards in Ireland and can serve as a foundation for broader application in primary care, community, and public health settings.

## Introduction

This review explores how dashboards are developed within the health care sector and which guidelines, if any, steer such development. Drawing insights from international research, this review ultimately aims to identify best practices and demonstrate how visual tools can enhance understanding, decision-making, and communication.

Dashboards are electronic displays that usually combine multiple visualizations to enable users to simultaneously access and interact with information on related topics [1]. Ideally, dashboards should support rapid understanding and reduce cognitive load, a key consideration in high-stress health care environments where time-sensitive decisions are paramount [2]. As visual dashboards can significantly improve decision-making [3], they should enable health care professionals to efficiently and effectively identify care challenges and progress. Health organizations use dashboards for different purposes, such as management and oversight, as well as for monitoring and improving direct patient care. Dashboards can also help improve teamwork and communication among people with different roles, expertise, and schedules [1].

Data visualization refers to the process of converting complex datasets into visual formats, such as charts and graphs. By facilitating clearer and more immediate insights, effective data visualizations have the potential to significantly improve information sharing among health care professionals, leading to enhanced collaborative decision-making [4]. This is supported by Lurie and Mason [5], who highlight that appropriately designed data visualizations not only improve comprehension but also enhance retention of critical information. Similarly, international studies have found that well-designed data visualization tools can reduce medical errors and support more accurate clinical decisions, particularly when used to communicate complex patient data [6].

Several frameworks for data visualization and dashboard design exist outside of health care and provide useful starting points. The nested model by Munzner [7] describes levels of design decision-making, from domain problems to visual encoding, emphasizing alignment between user tasks and visualization choices. The dashboard design principles by Few [8] offer practical guidance on clarity, simplicity, and information density, primarily targeted at business contexts. Human-computer interaction (HCI) standards, such as ISO 9241-210 [9], similarly emphasize usability, accessibility, and iterative user-centered design. These frameworks are valuable, but their application to health care contexts is limited. They often neglect the complexities of health care data, such as interoperability, sensitive patient information, and the unique cognitive demands of clinical environments.

Previous reviews of health care dashboards have advanced the field but remain constrained in several ways. For example, a scoping review by Helminski et al [10] found that few included studies used usability testing or formal theoretical frameworks for design or evaluation. The systematic review of hospital dashboards by Rabiei and Almasi [11] identified both functional and nonfunctional requirements but reported that many studies lacked empirical evaluation and had limited detail on implementation or integration with existing systems. As a result, existing evidence has not yet been consolidated into a structured, theoretically grounded framework that can guide the design of health care dashboards across settings.

To address this gap, we undertook an international scoping review of health care dashboard literature. Our aim was not only to map and catalog existing practices but also to organize them into a cohesive framework that bridges theory and practice. Building on previous work, our contribution is to consolidate existing evidence into 4 core pillars of dashboard design (approach, content, behavior, and adoption), which provide a foundation for more consistent, evidence-informed dashboard development in health care while remaining adaptable to diverse contexts.

## Methods

### Study Design

This study was conducted as a scoping review, following the framework proposed by Arksey and O’Malley [12] and enhanced by Levac et al [13]. Scoping reviews are particularly suited to mapping the existing literature in areas where guidelines are not well established, identifying key concepts, and synthesizing recurring practices. Our objective was to explore the international literature on health care dashboard design and identify common practices. As such, we did not perform formal quality appraisals, which is consistent with scoping review methodology. We report this review in accordance with the PRISMA-ScR (Preferred Reporting Items for Systematic Reviews and Meta-Analyses extension for Scoping Reviews) checklist [14] (Multimedia Appendix 1).

### Needs Assessment

The initial proposal emerged from discussions between health professionals working on health service improvement initiatives and a data visualization expert. On the basis of a need to develop regional population profiles, a lack of clarity on effective data presentation was identified as an area that needed to be addressed.

### Identify the Research Question

The objective of this scoping review was to explore current international health care literature to identify existing guidelines or, in the absence of such guidelines, to identify common practices for health care dashboard design. The following research questions guided the search strategy: (1) What dashboards exist within (national) health systems that support decision-making? (2) Are there good practice guidelines for the creation of such dashboards? and (3) If not, are the design practices sufficiently described to enable the creation of dashboard design guidelines?

### Proposal Development and Approval

The questions and the overall time plan were discussed with the project team. Concurrently, discussions with other stakeholders involved in the development of health dashboards allowed the team to gather feedback regarding the potential impact of developing an evidence summary.

### Systematic Literature Search

We conducted an electronic search using PubMed, Embase, Scopus, and IEEE Xplore. The search strategy combined terms for dashboards and data visualization (eg, dashboard, data visualization, and visual analytics) with terms for health care and health systems (eg, health care and health infrastructure). Boolean operators and truncation were used to capture variations. Controlled vocabulary (eg, Medical Subject Headings [MeSH] terms such as data visualization and decision support systems) was included where applicable. These keywords and associated terms were based on the research questions and manual searches for papers about the topic. Searches were limited to English-language articles published between January 2014 and April 2024. The complete reproducible search strings for each database are provided in Multimedia Appendix 2.

### Screen and Select Relevant Literature

On completion of the electronic searches, the results were imported into Microsoft Excel. After removal of duplicates, titles and abstracts were screened by 2 independent reviewers. Conflicts or disagreements about selections were resolved by a third independent reviewer.

Eligibility criteria were defined to ensure that the review captured evidence most relevant to contemporary health care dashboard design. We limited the search to publications from 2014 onward, reflecting significant advances in data visualization technologies, health care information systems, and the use of dashboards in health service decision-making over the past decade. Only English-language publications were included due to resource constraints for translation. We included peer-reviewed research papers, systematic reviews, and dissertations or theses, as these sources often provide detailed methodological and design insights. In contrast, conference abstracts, commentaries, and opinion papers were excluded because they generally provide insufficient methodological detail or practical guidance. The specific inclusion and exclusion criteria are summarized in Table 1.

**Table 1:** Eligibility criteria used for the screening of studies.

	Inclusion criteria	Exclusion criteria
Population	Data visualization and data dashboards	Clinical visualizations and clinical visual aids
Context	All health care settings and any country or health system	None
Types of evidence sources	Research papers, systematic reviews, and dissertations or theses (including empirical findings and author-reported practices)	Conference abstracts and commentaries or opinion papers
Languages	English	Other than English
Time	2014 to 2024	Publications before 2014

Consistent with scoping review methodology and PRISMA-ScR guidance, no formal risk of bias or quality appraisal of included studies was undertaken, as our aim was to map the characteristics and range of existing evidence rather than to assess study validity.

After the initial screening of titles and abstracts, the selected studies were reviewed in full by 2 authors, and any disagreements were resolved by a third reviewer.

A data charting table summarizing key characteristics of the included studies is provided in Multimedia Appendix 3. The table also indicates the extent to which each study described guidelines and specifies which sections of the paper were used for reflexive thematic analysis (RTA) [15].

### Identify Pillars From Included Studies

After the full-text screening was complete, the guidelines and practices described were extracted into an Excel sheet. Using RTA, the first author (HV) coded each described practice with a descriptive keyword (eg, *user engagement* or *data quality*) and iteratively grouped related codes into broader conceptual clusters. These clusters were reviewed and discussed by all team members (HV, SB, and CC), leading to the identification of four overarching themes (pillars):

1. Approach—the *methods* and *mindset* that shape the design process
2. Content—the *type of information* that should be provided and displayed
3. Behavior—the *manner of interaction* with the information (access, user-friendliness, and navigation)
4. Adoption—the *practices* that support adoption and ongoing engagement

Multimedia Appendix 4 lists the second-level keywords that informed each pillar. The analysis was conducted in line with the interpretive and reflexive orientation of RTA, emphasizing depth of understanding over coder agreement or reproducibility.

### Produce Reports

This paper presents the first report arising from this search. Future papers will detail how the identified pillars and associated practices have been contextualized in the Irish context and describe the overall impact of this initiative.

### Ongoing Follow-Up and Dialogue

The pillars and associated practices will inform a series of workshops with users and stakeholders to communicate and contextualize the findings.

## Results

**Figure 1:**
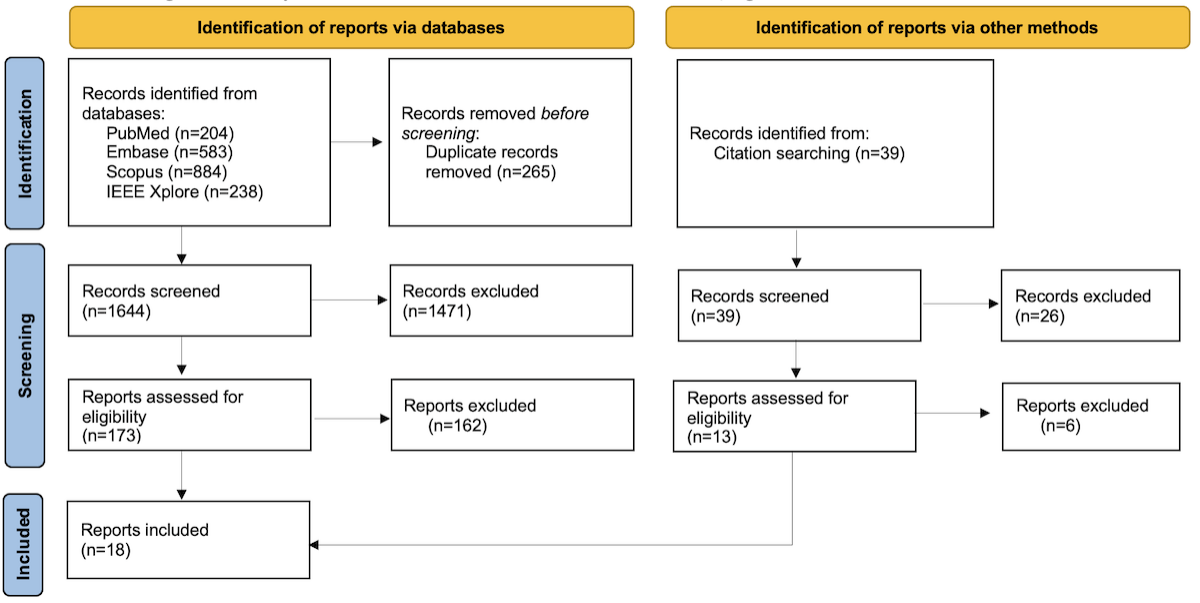
PRISMA-ScR (Preferred Reporting Items for Systematic Reviews and Meta-Analyses extension) flow diagram showing selection of relevant papers [16].

After removing duplicates, a total of 1644 potentially relevant reports were identified. Two reviewers (HV and SB) screened the abstracts of the selected papers, while a third reviewer (CC) resolved disagreements between the 2 reviewers. A total of 1471 papers were excluded, while 173 were selected for full-text review. A citation search of the selected papers led to a further 39 records, which were screened as outlined earlier. This added another 13 papers to the 173 papers to be included in the full-text review. After assessing against the eligibility criteria, a total of 18 reports were included in the synthesis of evidence. The entire selection process is represented in a PRISMA-ScR flow diagram (Figure 1; full study characteristics and extracted data are provided in Multimedia Appendix 3) [16].

A total of 18 studies were included, with the majority focused on hospital settings (n=13, 72.2%). Few studies addressed national or public dashboards, and dashboards implemented at the community level were notably underrepresented, indicating a gap in evidence for nonhospital contexts. Two papers were review articles. Three papers focused on the development of national dashboards or the integration of a mobile app with a dashboard. Studies based in UK hospitals were the focus in 4 papers, while studies in the United States and the Netherlands were the setting for 2 papers each. The remainder included 1 study each in Australia, Iran, Portugal, Somalia, South Africa, South Korea, and Switzerland. Three papers did not specify the country in which the study was conducted.

In total, 4 papers [17-20] explicitly described comprehensive guidelines for dashboard development from start to finish, highlighting structured and replicable approaches*,* while an additional 9 papers [11,21-28] presented practices addressing specific aspects, requirements, or phases of dashboard design. The remaining 5 papers [29-33] either outlined specific design goals or described how dashboards were developed in practice but did not offer development practices that could inform broader or generalizable approaches to dashboard design. Overall, most implementations appeared ad hoc rather than following a structured framework, which may limit reproducibility and generalizability. Notably, half of the studies (9/18, 50%) focused primarily on evaluating dashboards after their creation, rather than explicitly detailing robust design principles during development.

Common practices identified across studies included user involvement in design and evaluation [17,28]; use of relevant and actionable metrics [25]; attention to data quality, including data accuracy and timeliness [30]; usability and interactivity [33]; and integration into existing workflows to avoid overburdening staff [20].

To illustrate, van de Baan et al [17] in the Netherlands developed quality and safety dashboards through a 5-stage participatory process with 13 health professionals across 2 care pathways. Their structured, clinician-led approach demonstrated how iterative co-design can enhance ownership and ensure dashboards address real-world needs (pillar: approach). Randell et al [25], drawing on National Health Service clinical audit data, highlighted challenges in selecting indicators and ensuring their accuracy, translating these into clear requirements for dashboards that can support decision-making “from ward to board” (pillar: content). Loorak et al [33] introduced TimeSpan, a dashboard for stroke care pathways, where interactivity enabled clinicians to generate new insights and explore patterns beyond static reporting (pillar: behavior). Finally, Weggelaar-Jansen et al [20] showed that sustainable dashboard adoption required embedding dashboards into governance routines and review meetings, not just technical development (pillar: adoption).

Contrasts and conflicts across settings were notable: hospital dashboards prioritized clinical indicators and immediate operational utility, whereas public dashboards emphasized transparency, accessibility, and stakeholder accountability. Community dashboards were minimally represented, suggesting that practical guidance for these contexts is lacking. Furthermore, divergences in design philosophy emerged: some studies emphasized highly interactive dashboards with rich visualizations, while others favored static simplicity to reduce cognitive load. Similarly, governance and sustainability considerations varied: certain implementations benefited from strong institutional buy-in, whereas others struggled to maintain dashboards over time. These differences highlight potential tensions between usability, engagement, and sustainability and underscore that no single approach fits all contexts.

Methodologically, studies differed in both their analytic rigor and their use of visualization techniques. Hospital-based dashboards typically adopted iterative or co-design methods, enabling clinical validation and rapid refinement of metrics [17,18], whereas national or public-facing dashboards tended to rely on top-down technical builds using aggregated data [26]. These methodological differences shaped the visualization choices observed: co-designed hospital dashboards favored targeted, high-resolution displays with limited indicator sets, while public health dashboards prioritized breadth, using simplified or static visuals to maximize accessibility. Studies using participatory design generally reported stronger usability and contextual fit, while purely technical implementations often struggled with user engagement or indicator relevance.

Context also influenced implementation and sustainability. Hospital dashboards were typically embedded within established governance structures, allowing integration into performance review meetings and continuous data validation [20]. In contrast, public dashboards faced challenges of data standardization, cross-agency coordination, and long-term ownership, which limited ongoing updates. Methodological variation, therefore, reflected contextual constraints: where clinical workflows supported routine data collection, interactive and iterative designs thrived; where data pipelines were diffuse, design flexibility was restricted. These contrasts illustrate that methodological rigor and stakeholder proximity are decisive factors in determining dashboard longevity and real-world impact.

More than half of the papers (11/18, 61.1%) identified evaluation, appropriate visual encoding [7,34], and user involvement as key to developing a dashboard. Other key practices included modularity (the ability to add or delete components) and data quality (including timeliness and accuracy). Interactivity and the need to avoid adding to staff’s existing workload were mentioned by 6 papers (33.3%).

**Figure 2:**
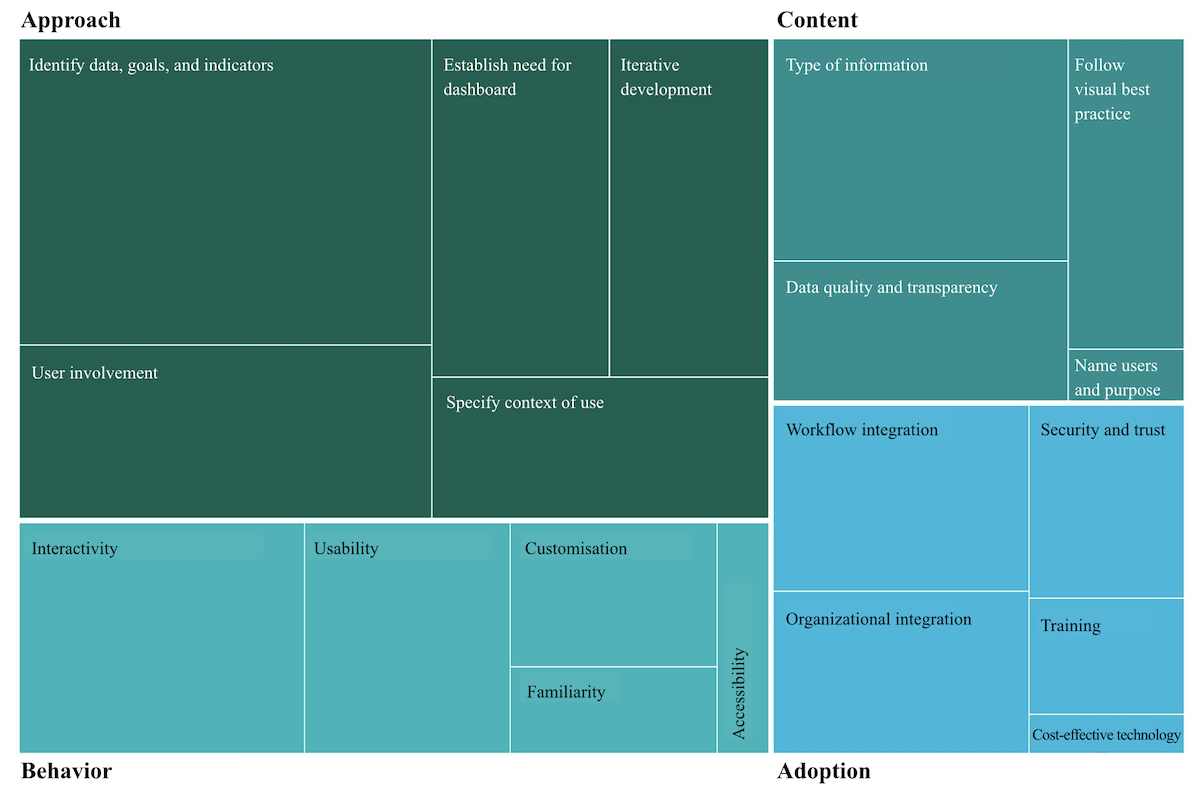
Key terms grouped by pillar: approach, content, behaviour and adoption.

The secondary analysis (RTA) resulted in 4 key themes (pillars) being defined: “approach,” “content,” “behavior,” and “adoption.” Most practices supported the “approach” pillar (33 unique mentions) (Table 2), while practices for “behavior” were comparatively few (15 unique mentions; Figure 2).

**Table 2:** Illustrative contributions of included studies to each pillar.

Pillar	Illustrative contributions (examples)
Approach	Participatory and iterative design approaches were reported in several studies [17,18,28], emphasizing co-design and ownership.
Content	Indicator selection and data quality were central concerns, as highlighted by Randell et al [25] on National Health Service clinical audits, Christen et al [32] on modular EMS dashboards, and Ivanković et al [26] on transparency in global COVID-19 dashboards.
Behavior	Usability and interactivity were highlighted in dashboards such as TimeSpan for stroke care [33], population health frameworks [16], and productivity dashboards [31].
Adoption	Sustainable uptake depended on workflow integration and governance structures, as described by Weggelaar-Jansen et al [20], Ahn et al [30], and Kamadjeu and Gathenji [27].

This finding underscores that while dashboard design is frequently addressed, critical attention to adoption, behavior change, and context-specific implementation is limited, suggesting gaps in translating design principles into practical impact.

## Discussion

### Overview

This review provides an insight into current practices in health care dashboard design. Our findings reveal a significant gap in the use of established guidelines or frameworks during the dashboard design process. While there is broad agreement on some core practices such as evaluation and user involvement, other areas such as data security or staff training are rarely mentioned. These gaps may arise from the reactive and ad hoc nature of dashboard implementation in response to immediate operational pressures, rather than from a lack of awareness of best practices. Moreover, our synthesis highlights how design priorities differ across health care contexts, reflecting the specific needs and constraints of each setting. Understanding these practices provides a foundation for mapping observed patterns to established visualization and HCI frameworks, which underpins the subsequent synthesis of the 4 pillars: approach, content, behavior, and adoption.

### Anchoring the 4 Pillars in Established Theory

#### Theoretical Alignment With Visualization

Each of the 4 pillars aligns with recognized frameworks in visualization and HCI. Approach reflects principles of user-centered and participatory design, emphasizing stakeholder involvement and iterative cocreation (ISO 9241-210 [9]). Content is consistent with the nested model by Munzner [7] and the semiology by Bertin [34], underscoring the importance of selecting actionable metrics and applying effective visual encoding. Behavior draws on HCI principles of usability [35] and the concept of affordances by Norman [36], highlighting interactivity, customization, and accessibility. Finally, adoption resonates with sociotechnical theories such as the technology acceptance model [37] and normalization process theory [38]. By mapping inductively derived themes to these theories, the 4 pillars provide a bridge between conceptual models and applied health care practice, ensuring the framework is both empirically grounded and theoretically anchored.

These theoretical frameworks were not used to generate the pillars but were applied retrospectively as an interpretive lens to examine their coherence and alignment with established concepts in visualization, usability, and sociotechnical theory. This approach ensures that the 4 pillars remain inductively grounded while being conceptually validated against recognized theoretical models.

#### Pillar 1: Approach

This pillar includes the methods and mindsets that shape the dashboard design process. It focuses strongly on involving and engaging end users and stakeholders in defining needs, iterative cocreation, and a review of existing systems to ensure that the best solution is put forward for development.

#### Pillar 2: Content

This pillar describes the type of information a dashboard should provide and how it should be displayed. It includes the need for relevant and actionable metrics and an examination of data accuracy and quality. Content should be presented following visual best practices (eg, using percentages instead of raw numbers, eliminating visual noise, and highlighting key signals) to ensure clarity and support decision-making.

#### Pillar 3: Behavior

Behavior includes practices related to how users interact with the dashboard. This includes interactivity to support engagement and exploration, as well as usability and accessibility considerations to accommodate diverse levels of data literacy.

#### Pillar 4: Adoption

This pillar focuses on practices that support adoption both by immediate users and the wider organization, thereby ensuring longevity and sustainability. Practices such as the ability to customize the dashboard, workflow integration to minimize additional staff workloads, broader integration into the organizational culture through stakeholder engagement, and continuous improvements help to ensure that the dashboard becomes part of daily operations. Attention to cost-effective tools, data security, privacy, and continuous evaluation further supports sustained adoption.

### Principal Findings

A key insight from our review is that 50% (9/18) of studies focused on evaluating dashboards after their creation rather than incorporating robust design principles during development. Evaluation efforts often highlighted issues with usability, data integration, and user engagement, underscoring the importance of proactive design strategies to address these challenges upfront. Ad hoc development and inconsistent application of design principles were common, contributing to persistent gaps in effective dashboard implementation. Adoption-related factors, such as governance, training, and sustainability, were rarely addressed, despite their known importance for long-term use.

Our analysis also demonstrates that context shapes dashboard priorities: hospitals often emphasize operational and clinical metrics to support immediate decision-making, while publicly accessible dashboards tend to prioritize transparency and stakeholder communication. Community and population health dashboards were scarce, which may explain the limited guidance on integrating diverse data sources or engaging broader stakeholder groups. Conflicting design priorities were also noted: some studies emphasized highly interactive dashboards to enhance engagement, while others favored simpler, static dashboards to reduce cognitive load or resource requirements. Technological, governance, and sustainability challenges were frequently overlooked, particularly in nonhospital or community settings.

Furthermore, while concepts such as user involvement and iterative refinement were mentioned, they were not consistently applied, leaving gaps in achieving effective, user-centered dashboards.

Taken together, the 4 pillars summarize a practical set of considerations for dashboard design and adoption. They provide a lens for assessing both technical and sociotechnical aspects, from user-centered approaches and data quality to usability, governance, and sustainability. For practice, this highlights embedding dashboards into workflows with training and stakeholder engagement. For policy, investment is required in the sociotechnical infrastructure to ensure dashboards are effective and sustainable beyond immediate returns.

### Strengths and Limitations

This review contributes to the understanding of best practices in health care dashboard design by synthesizing existing literature and identifying key themes. The integration of theory strengthens the framework and allows critical evaluation of divergences and gaps. Limitations include scope and selection bias, lack of empirical validation, and the overrepresentation of hospital settings. Future research should test the 4 pillars in underrepresented contexts, such as primary care, community health, and public dashboards, and evaluate impacts on usability, adoption, and decision-making.

### Implications for Ireland

Although international in scope, these findings provide an evidence base for the next phase of work in the Irish health care system. The 4 pillars will serve as the starting point for a consensus building process with clinicians, public health professionals, service planners, policymakers, IT specialists, and patient or community representatives. This process will contextualize international practices to Irish data infrastructures, governance, and regulatory frameworks, ensuring that future dashboards are both evidence informed and locally relevant.

### Future Work

This review represents an initial step toward establishing structured, evidence-based guidelines for health care dashboard design, synthesizing international best practices into the 4 pillars: approach, content, behavior, and adoption. Building on the findings of this international review, the next phase of this work focuses on consensus building and co-design within the Irish health care system. Specifically, the 4 pillars will provide the starting point for structured discussions with Irish stakeholders, ensuring that international evidence is explicitly adapted to local infrastructure, governance, and regulatory requirements. By engaging a diverse range of stakeholders—including public health professionals; service planners; clinicians; IT specialists; policymakers; and public, patient, and community partners—we will refine the practices under each of the 4pillars into actionable, context-specific guidelines. For example, the practice of ensuring data quality (pillar: content) will be mapped to current data sources within Ireland, their interoperability, and Ireland-specific data protection principles (eg, General Data Protection Regulation [39]), illustrating how international guidance informs practical national implementation.

Through this iterative process, the resulting guidelines will be globally informed; locally applicable; and co-designed to foster equity, accessibility, and usability.

Several areas also warrant further investigation to strengthen the evidence base supporting the 4 pillars. First, empirical evaluation of the 4 pillars across diverse health care settings, including primary care, community health, and public-facing dashboards, could test their impact on usability, adoption, and decision-making. Second, the 4 pillars could be translated into practical tools, such as checklists, design templates, or evaluation criteria, for use by health care organizations and developers. Third, comparative studies with other sectors (eg, business intelligence and education) may provide insights into transferable practices and highlight areas requiring health care–specific adaptation.

### Conclusions

This review is an essential first step in understanding the global landscape of health care dashboards and provides a foundation for contextualization in Ireland. As outlined earlier, we have identified a significant gap, namely, a lack of standardized principles that could guide the design of dashboards in the health care sector. Arising from this review, we have defined 4 main pillars with associated key practices. These 4 pillars, derived from the international literature, will guide the upcoming consensus building process in Ireland and ensure that international evidence informs locally relevant guidelines. They will serve as the foundation for creating a comprehensive framework for health care data visualization in Ireland.

Ultimately, this phased approach from international review to consensus building and framework development ensures a solid foundation of evidence and co-design for transforming the design of data dashboards in the Irish health care system. By enabling dashboards that are not only functional but also user-friendly and impactful, this work supports improved decision-making, resource allocation, and health outcomes for all stakeholders.

## Appendix

Multimedia Appendix 1 PRISMA-ScR checklist.

Multimedia Appendix 2 Electronic search report.

Multimedia Appendix 3 Characteristics of reports included.

Multimedia Appendix 4 Overview of coding for reflexive thematic analysis.

Multimedia Appendix 5 Reports mapped to pillars.

## Abbreviations

MeSH: Medical Subject Headings
PRISMA-ScR: Preferred Reporting Items for Systematic Reviews and Meta-Analyses extension for Scoping Reviews
RTA: reflexive thematic analysis
