# Supplementary material for: Design Practices for Data Dashboards in Health Care: Scoping Review
Source: J Med Internet Res. 2026 Feb 25;28:e77361. doi: 10.2196/77361 (PMC12980066; doi:10.2196/77361)
Supplement: Multimedia Appendix 2 [file jmir_v28i1e77361_app2.docx]

Appendix 2. Evidence search report in electronic databases

| **Electronic search report No. 1** | |
| --- | --- |
| **Electronic database** | Medline |
| **Platform** | PubMed |
| **Date of search** | 27-04-2024 |
| **Range of date** | 2014 to 2024 |
| **Restriction of language** | English |
| **Other limits** | None |
| **Search strategy** | (("Dashboard*"[Title/Abstract] OR "Data Visualization"[Title/Abstract]  OR "Data Visualisation"[Title/Abstract] OR "Visual Analytics"[Title/Abstract] OR "Visual Tool*"[Title/Abstract] OR "Visual Support"[Title/Abstract] OR "Visual Aid"[Title/Abstract])  OR "Data Visualization"[MeSH Terms])  AND  ("Healthcare"[Title/Abstract] OR "Health System*"[Title/Abstract]  OR "Health Infrastructure"[Title/Abstract] OR "Healthcare Management"[Title/Abstract] OR "Health Services"[MeSH Terms])  AND  ("Guidelines"[Title/Abstract] OR "Good Practice"[Title/Abstract] OR "Decision-making"[Title/Abstract] OR "Communication"[Title/Abstract] OR "Evidence-based Practice"[Title/Abstract] OR "Public Reporting"[Title/Abstract] OR "Computer-assisted Decision-making"[Title/Abstract] OR "Practice Guidelines as Topic"[MeSH Terms] OR "Decision Support Systems, Clinical"[MeSH Terms]) |
| **Number of references found** | 204 |
| **Electronic search report No. 2** | |
| **Electronic database** | Embase |
| **Platform** | Elsevier |
| **Date of search** | 01-05-2024 |
| **Range of date** | 2014 to 2024 |
| **Restriction of language** | English |
| **Other limits** | None |
| **Search strategy** | (('dashboard':ti,ab OR 'data visuali*ation':ti,ab OR 'visual analytics':ti,ab OR 'visualisation tool':ti,ab OR 'visual aid':ti,ab OR 'visual support':ti,ab)  OR 'data visualization'/exp)  AND  (('health care':ti,ab OR 'healthcare management':ti,ab OR 'health infrastructure':ti,ab OR 'health care quality':ti,ab OR 'health service*':ti,ab) OR 'health care'/exp)  AND  (('guideline*':ti,ab OR 'good practice':ti,ab OR 'decision-making':ti,ab  OR 'evidence-based practice':ti,ab OR 'communication':ti,ab OR 'public reporting':ti,ab OR 'decision support system':ti,ab) OR 'practice guideline'/exp OR 'decision support system'/exp) |
| **Number of references found** | 583 |
| **Electronic search report No. 3** | |
| **Electronic database** | SCOPUS |
| **Platform** | SCOPUS |
| **Date of search** | 01-05-2024 |
| **Range of date** | 2014 to 2024 |
| **Restriction of language** | English |
| **Other limits** | None |
| **Search strategy** | (TITLE-ABS-KEY(dashboard OR "data visuali*ation" OR "visual analytics" OR "visualisation tool" OR "visual aid" OR "visual support"))  AND  (TITLE-ABS-KEY("health*care" OR "health system*" OR "healthcare management" OR "health infrastructure" OR "health service*" OR "healthcare quality" OR "public reporting of quality data" OR "public reporting of healthcare data"))  AND  (TITLE-ABS-KEY("guideline*" OR "good practice" OR "communication" OR "decision-making" OR "evidence-based practice" OR "decision support system"))  **Filters applied:** Publication years 2014–2024; English language; document types = articles, conference papers, and book chapters. |
| **Number of references found** | 884 |
| **Electronic search report No. 4** | |
| **Electronic database** | IEEE Explore |
| **Platform** | IEEE |
| **Date of search** | 02-05-2024 |
| **Range of date** | 2014 to 2024 |
| **Restriction of language** | None |
| **Other limits** | None |
| **Search strategy** | (("dashboard" OR "data visuali*ation" OR "visual analytics" OR "visualisation tool" OR "visual aid" OR "visual support") IN Document Title)  AND  (("health*care" OR "healthcare management" OR "health service*" OR "healthcare quality" OR "public reporting of quality data" OR "public reporting of healthcare data" OR "health infrastructure") IN All Metadata)  AND  (("guideline*" OR "good practice" OR "communication" OR "decision-making" OR "evidence-based practice" OR "decision support system")  IN All Metadata) |
| **Number of references found** | 238 |
