# Supplementary material for: Design Practices for Data Dashboards in Health Care: Scoping Review
Source: J Med Internet Res. 2026 Feb 25;28:e77361. doi: 10.2196/77361 (PMC12980066; doi:10.2196/77361)
Supplement: Multimedia Appendix 4 [file jmir_v28i1e77361_app4.docx]

# Appendix 4. Reflexive Thematic Analysis – Mention of key terms

| **n** | **ID record** | **Approach** | | | |  | **Content** | | | | **Behavior** | | | | | **Adoption** | | | | |
| --- | --- | --- | --- | --- | --- | --- | --- | --- | --- | --- | --- | --- | --- | --- | --- | --- | --- | --- | --- | --- |
|  |  | Establish need | Identify data, goals etc. | Iterative development | User involvement | Specify context of use | Data quality | Visual best practice | Name users and purpose | Type of information | Accessibility | Customization | Familiarity | Interactivity | Usability | Cost-effective technology | Organiszational integration | Security and trustr | Training | Workflow integration |
| [29] | Ratwani 2014 | x | x | x | x |  |  |  |  | x | x |  |  | x | x |  |  |  | x | x |
| [30] | Ahn 2021 | x | x |  | x | x |  |  |  |  |  |  |  |  | x |  |  |  |  |  |
| [21] | Lee 2016 | x |  | x |  |  |  |  |  |  |  |  |  |  |  |  |  |  |  |  |
| [22] | Alvarado 2021 |  |  | x |  | x |  |  |  |  |  |  |  |  |  |  | x |  |  |  |
| [23] | Bunting 2017 |  | x |  | x | x | x | x |  | x |  |  |  | x | x |  |  |  |  | x |
| [24] | Fazaeli 2021 | x | x | x | x | x |  | x |  |  |  |  |  |  |  |  |  |  |  |  |
| [31] | Pestana 2020 | x | x | x |  |  | x | x |  | x |  |  |  | x | x |  | x |  |  |  |
| [17] | van de Baan 2023 | x |  | x | x |  |  |  |  |  |  |  |  |  |  |  | x |  | x | x |
| [25] | Randell 2020 |  | x |  | x |  | x |  |  | x |  | x | x | x |  |  | x |  |  |  |
| [26] | Ivanković 2021 | x | x |  |  | x | x |  | x | x |  |  |  | x |  |  |  |  |  |  |
| [18] | Elshehaly 2021 | x |  | x | x | x | x |  |  | x |  |  |  | x |  |  |  | x |  | x |
| [32] | Christen 2020 |  |  |  |  |  |  | x |  | x |  |  | x |  | x |  |  |  |  |  |
| [27] | Kamadjeu 2017 | x | x |  |  |  |  |  |  |  |  | x |  |  |  | x |  |  |  | x |
| [19] | Concannon 2019 |  | x |  | x | x |  |  |  | x |  | x |  | x |  |  | x | x |  |  |
| [20] | Weggelaar-Jansen 2018 |  | x |  | x |  | x | x |  | x |  | x |  | x | x |  | x |  |  | x |
| [28] | Khanbhai 2022 | x |  | x | x |  |  |  |  | x |  |  |  | x | x |  |  | x |  | x |
| [11] | Rabiei 2022 |  | x |  | x | x |  |  |  | x |  | x |  | x | x |  | x | x | x | x |
| [33] | Loorak 2016 |  |  | x |  |  | x | x |  |  | x |  | x | x |  |  |  | x |  |  |

**References**

[29] Ratwani RM, Fong A. 'Connecting the dots': leveraging visual analytics to make sense of patient safety event reports. J Am Med Inform Assoc. 2015 Mar;22(2):312-7. doi: 10.1136/amiajnl-2014-002963. Epub 2014 Oct 21. PMID: 25336592; PMCID: PMC11749149.

[30] Ahn E, Liu N, Parekh T, Patel R, Baldacchino T, Mullavey T, Robinson A, Kim J. A Mobile App and Dashboard for Early Detection of Infectious Disease Outbreaks: Development Study. JMIR Public Health Surveill. 2021 Mar 9;7(3):e14837. doi: 10.2196/14837. PMID: 33687334; PMCID: PMC7988388.

[21] Lee K, Jung SY, Hwang H, Yoo S, Baek HY, Baek RM, Kim S. A novel concept for integrating and delivering health information using a comprehensive digital dashboard: An analysis of healthcare professionals' intention to adopt a new system and the trend of its real usage. Int J Med Inform. 2017 Jan;97:98-108. doi: 10.1016/j.ijmedinf.2016.10.001. Epub 2016 Oct 6. PMID: 27919400.

[22] Alvarado N, McVey L, Elshehaly M, Greenhalgh J, Dowding D, Ruddle R, Gale CP, Mamas M, Doherty P, West R, Feltbower R, Randell R. Analysis of a Web-Based Dashboard to Support the Use of National Audit Data in Quality Improvement: Realist Evaluation. J Med Internet Res. 2021 Nov 23;23(11):e28854. doi: 10.2196/28854. PMID: 34817384; PMCID: PMC8663683.

[23] Bunting RF Jr, Siegal D. Developing risk management dashboards using risk and quality measures: A visual best practices approach. J Healthc Risk Manag. 2017 Oct;37(2):8-28. doi: 10.1002/jhrm.21287. Epub 2017 Sep 28. PMID: 28960593.

[24] Fazaeli S, Khodaveisi T, Vakilzadeh AK, Yousefi M, Ariafar A, Shokoohizadeh M, Mohammad-Pour S. Development, Implementation, and User Evaluation of COVID-19 Dashboard in a Third-Level Hospital in Iran. Appl Clin Inform. 2021 Oct;12(5):1091-1100. doi: 10.1055/s-0041-1740188. Epub 2021 Dec 8. PMID: 34879405; PMCID: PMC8654579.

[31] Pestana M, Pereira R, Moro S. Improving Health Care Management in Hospitals Through a Productivity Dashboard. J Med Syst. 2020 Mar 12;44(4):87. doi: 10.1007/s10916-020-01546-1. PMID: 32166499.

[17] van de Baan FC, Lambregts S, Bergman E, Most J, Westra D. Involving Health Professionals in the Development of Quality and Safety Dashboards: Qualitative Study. J Med Internet Res. 2023 Jun 12;25:e42649. doi: 10.2196/42649. PMID: 37307058; PMCID: PMC10337379.

[25] Randell R, Alvarado N, McVey L, Ruddle RA, Doherty P, Gale C, Mamas M, Dowding D. Requirements for a quality dashboard: Lessons from National Clinical Audits. AMIA Annu Symp Proc. 2020 Mar 4;2019:735-744. PMID: 32308869; PMCID: PMC7153077.

[26] Ivanković D, Barbazza E, Bos V, Brito Fernandes Ó, Jamieson Gilmore K, Jansen T, Kara P, Larrain N, Lu S, Meza-Torres B, Mulyanto J, Poldrugovac M, Rotar A, Wang S, Willmington C, Yang Y, Yelgezekova Z, Allin S, Klazinga N, Kringos D. Features Constituting Actionable COVID-19 Dashboards: Descriptive Assessment and Expert Appraisal of 158 Public Web-Based COVID-19 Dashboards. J Med Internet Res. 2021 Feb 24;23(2):e25682. doi: 10.2196/25682. PMID: 33577467; PMCID: PMC7906125.

[18] Elshehaly M, Randell R, Brehmer M, McVey L, Alvarado N, Gale CP, Ruddle RA. QualDash: Adaptable Generation of Visualisation Dashboards for Healthcare Quality Improvement. IEEE Trans Vis Comput Graph. 2021 Feb;27(2):689-699. doi: 10.1109/TVCG.2020.3030424. Epub 2021 Jan 28. PMID: 33048727.

[32] Christen OM, Mösching Y, Müller P, Denecke K, Nüssli S. Dashboard Visualization of Information for Emergency Medical Services. Stud Health Technol Inform. 2020 Nov 23;275:27-31. doi: 10.3233/SHTI200688. PMID: 33227734.

[27] Kamadjeu R, Gathenji C. Designing and implementing an electronic dashboard for disease outbreaks response - Case study of the 2013-2014 Somalia Polio outbreak response dashboard. Pan Afr Med J. 2017 Jun 22;27(Suppl 3):22. doi: 10.11604/pamj.supp.2017.27.3.11062. PMID: 29296157; PMCID: PMC5745940.

[19] Concannon D, Herbst K, Manley E. Developing a Data Dashboard Framework for Population Health Surveillance: Widening Access to Clinical Trial Findings. JMIR Form Res. 2019 Apr 4;3(2):e11342. doi: 10.2196/11342. PMID: 30946016; PMCID: PMC6470464.

[20] Weggelaar-Jansen AMJWM, Broekharst DSE, de Bruijne M. Developing a hospital-wide quality and safety dashboard: a qualitative research study. BMJ Quality & Safety 2018;27:1000-1007.

[28] Khanbhai M, Symons J, Flott K, Harrison-White S, Spofforth J, Klaber R, Manton D, Darzi A, Mayer E. Enriching the Value of Patient Experience Feedback: Web-Based Dashboard Development Using Co-design and Heuristic Evaluation. JMIR Hum Factors. 2022 Feb 3;9(1):e27887. doi: 10.2196/27887. PMID: 35113022; PMCID: PMC8855286.

[11] Rabiei R, Almasi S. Requirements and challenges of hospital dashboards: a systematic literature review. BMC Med Inform Decis Mak. 2022 Nov 8;22(1):287. doi: 10.1186/s12911-022-02037-8. PMID: 36348339; PMCID: PMC9644506.

[33] Loorak MH, Perin C, Kamal N, Hill M, Carpendale S. TimeSpan: Using Visualization to Explore Temporal Multi-dimensional Data of Stroke Patients. IEEE Trans Vis Comput Graph. 2016 Jan;22(1):409-18. doi: 10.1109/TVCG.2015.2467325. Epub 2015 Aug 12. PMID: 26390482.
